# The Repertoire of ICE in Prokaryotes Underscores the Unity, Diversity, and Ubiquity of Conjugation

**DOI:** 10.1371/journal.pgen.1002222

**Published:** 2011-08-18

**Authors:** Julien Guglielmini, Leonor Quintais, Maria Pilar Garcillán-Barcia, Fernando de la Cruz, Eduardo P. C. Rocha

**Affiliations:** 1 Institut Pasteur, Microbial Evolutionary Genomics, Département Génomes et Génétique, Paris, France; 2 CNRS, URA2171, Paris, France; 3 Departamento de Biología Molecular e Instituto de Biomedicina y Biotecnología de Cantabria (IBBTEC), Universidad de Cantabria-CSIC-SODERCAN, Santander, Spain; Universidad de Sevilla, Spain

## Abstract

Horizontal gene transfer shapes the genomes of prokaryotes by allowing rapid acquisition of novel adaptive functions. Conjugation allows the broadest range and the highest gene transfer input per transfer event. While conjugative plasmids have been studied for decades, the number and diversity of integrative conjugative elements (ICE) in prokaryotes remained unknown. We defined a large set of protein profiles of the conjugation machinery to scan over 1,000 genomes of prokaryotes. We found 682 putative conjugative systems among all major phylogenetic clades and showed that ICEs are the most abundant conjugative elements in prokaryotes. Nearly half of the genomes contain a type IV secretion system (T4SS), with larger genomes encoding more conjugative systems. Surprisingly, almost half of the chromosomal T4SS lack co-localized relaxases and, consequently, might be devoted to protein transport instead of conjugation. This class of elements is preponderant among small genomes, is less commonly associated with integrases, and is rarer in plasmids. ICEs and conjugative plasmids in proteobacteria have different preferences for each type of T4SS, but all types exist in both chromosomes and plasmids. Mobilizable elements outnumber self-conjugative elements in both ICEs and plasmids, which suggests an extensive use of T4SS in *trans*. Our evolutionary analysis indicates that switch of plasmids to and from ICEs were frequent and that extant elements began to differentiate only relatively recently. According to the present results, ICEs are the most abundant conjugative elements in practically all prokaryotic clades and might be far more frequently domesticated into non-conjugative protein transport systems than previously thought. While conjugative plasmids and ICEs have different means of genomic stabilization, their mechanisms of mobility by conjugation show strikingly conserved patterns, arguing for a unitary view of conjugation in shaping the genomes of prokaryotes by horizontal gene transfer.

## Author Summary

Some mobile genetic elements spread genetic information horizontally between prokaryotes by conjugation, a mechanism by which DNA is transferred directly from one cell to the other. Among the processes allowing genetic transfer between cells, conjugation is the one allowing the simultaneous transfer of larger amounts of DNA and between the least related cells. As such, conjugative systems are key players in horizontal transfer, including the transfer of antibiotic resistance to and between many human pathogens. Conjugative systems are encoded both in plasmids and in chromosomes. The latter are called Integrative Conjugative Elements (ICE); and their number, identity, and mechanism of conjugation were poorly known. We have developed an approach to identify and characterize these elements and found more ICEs than conjugative plasmids in genomes. While both ICEs and plasmids use similar conjugative systems, there are remarkable preferences for some systems in some elements. Our evolutionary analysis shows that plasmid conjugative systems have often given rise to ICEs and vice versa. Therefore, ICEs and conjugative plasmids should be regarded as one and the same, the differences in their means of existence in cells probably the result of different requirements for stabilization and/or transmissibility of the genetic information they contain.

## Introduction

Prokaryotes, both bacteria and archaea, have remarkably plastic genomes because they can acquire genetic information at high rates by horizontal transfer from other prokaryotes. This allows them to adapt rapidly to specific niches and results in large differences in gene repertoires among closely related strains [1]–[3]. Three major mechanisms allow gene transfer: natural transformation, transduction and conjugation. Natural transformation is controlled by the receptor cell and mostly implicated in DNA transfer within species leading to allelic recombination [4]. Both transduction and conjugation are more invasive, since the recipient has little control over both processes which change gene repertoires dramatically and allow transfer between distant lineages. Conjugation, in particular, can lead to the transfer of very large fractions of genomes and even entire chromosomes in one single event [5], [6]. Several studies suggest that conjugation is the preponderant mechanism of horizontal gene transfer between distant lineages [7], [8]. Such cross-clade transfer might be at the origin of the rapid spread of antibiotic resistance through most major lineages of bacterial pathogens in the last few decades [2], [9], [10]. Conjugative elements are also known for encoding other adaptive traits such as toxins, transporters and many secreted proteins including enzymes of industrial interest [11], [12].

**Figure 1:**
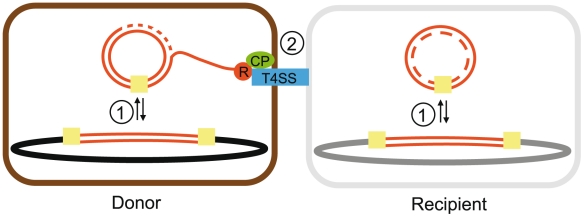
Scheme of some essential interactions in the process of ICEs movement. The integration/excision reaction (1) occurs by recombination across two recombination sites (yellow squares) located at the termini of the inserted element. As a result, a circular (most commonly non-replicating) DNA molecule is produced. Conjugation (2) is carried out by mobility systems. The relaxase (red circle) cleaves a specific site within *oriT*, and this step starts conjugation. The DNA strand that contains the relaxase protein covalently bound to its 5′-end is displaced by an ongoing conjugative DNA replication process (dotted lines). The relaxase interacts with the T4CP (green oval) and then with other components of the T4SS (blue rectangle). As a result, the relaxase-DNA complex is transported to the recipient cell [13]. Since ICEs are supposed not to replicate autonomously, the process terminates necessarily by integration of the transferred DNA circle in the recipient chromosome.

Conjugation involves a relaxase (MOB), which is the key element in a multiprotein DNA-processing complex, a type IV secretion system (T4SS) and a type IV coupling protein (T4CP) (reviewed recently in [13]) (Figure 1). The relaxase binds and nicks the DNA at the origin of transfer. The relaxase-DNA nucleoprotein complex is then coupled to the T4SS by the T4CP. The T4SS translocates the relaxase-DNA complex through the membrane of the donor cell delivering it to the cytoplasm of the recipient cell. The T4SS is a large complex of proteins spanning from the cytoplasm to the extracellular space, including an ubiquitous ATPase (VirB4 or TraU), a set of mating-pair formation (MPF) proteins (from a minimum of 12 to more than 20) that elaborate the transport channel, as well as a pilus that allows the attachment to the recipient cell and thereby the translocation of the relaxase-DNA complex. Protein homology of MPF genes allowed the clustering of all known proteobacterial T4SS into four groups [14], named after one model of each group, the vir system of the Ti plasmid (MPF_T_) [15], the F plasmid (MPF_F_) [16], the R64 IncI plasmid (MPF_I_) [17] and the integrative conjugative element (ICE) ICEHIN1056 (MPF_G_) [18]. For other taxonomic clades, the genes associated with the T4SS, apart from VirB4, the T4CP and the relaxase, are poorly characterized. Once the relaxase-DNA complex is in the recipient cell, the T4CP translocates the full DNA and the relaxase ligates the two ends of the DNA into a single circular molecule. At the final stage of the conjugation process, the element exists in ssDNA state in both cells and the hosts' replication machineries are recruited to replicate them to reconstitute the original dsDNA molecules [13]. A self-transmissible conjugative element must thus comprise three components: the relaxase, the T4CP, and the T4SS.

While most described conjugative systems are located in plasmids, the last decade has seen a growing interest in conjugative systems integrated in chromosomes (ICEs), which include the so-called “conjugative transposons” or “integrated conjugative plasmids” [19], [20]. The conjugation of ICEs is poorly documented but is generally assumed to resemble that of plasmids, with a preliminary step of excision with circularization and an additional final step of re-integration in the genome (Figure 1). For these steps, some ICEs encode supplementary genes resembling those of temperate phages, e.g. integrases of the lambda tyrosine-recombinase family [21], [22], which have led to their classification as “phage-like elements”. Other ICEs integrate in the chromosome, or excise from it, by using other tyrosine-recombinases [23], [24], DDE-transposases [25], serine-recombinases [26] or by homologous recombination with chromosomal copies of transposable elements [27], [28]. Contrary to plasmids, there is little evidence of ICEs replication in cells (but see, for instance, [29]) so it is often assumed that they cannot be stably maintained in an extra-chromosomal state [20]. While ICEs, by definition, are conjugative elements, many other mobile elements populate prokaryotic genomes. Integrative mobilizable elements (IMEs) do not code for a T4SS but can use one coded by other elements just like mobilizable plasmids [30], [31]. Genomic islands are integrative elements that can be mobilized by conjugation when they have compatible origins of transfer [32] or by integrating in conjugative elements [33]. Yet, like for non-mobilizable plasmids, the exact mechanism of mobility of most of these elements remains obscure [34]. Finally, some chromosomes encode T4SS that are not involved in conjugation but in other processes such as protein secretion and natural transformation [35], [36]. It has been suggested that these T4SS probably derived from ancestral conjugative systems [37].

The presence of an ICE can in principle be assessed by the observation of a conjugative T4SS within a chromosome. Since it is presently known how to class transmissible plasmids [14], it should be possible to do the same for ICEs. There are however important difficulties in this process. First, it is not known if all ICEs conjugate like plasmids. The family of conjugative elements of ICEHin1056 was proposed to exist exclusively as ICEs [18]. Even though a few rare conjugative plasmids of this family were subsequently identified [14], there might be other families exclusive to ICEs. Second, the presence in chromosomes of T4SS not used for conjugation may obscure the identification of conjugation systems if no relaxase is present at the locus. Third, the most conserved proteins involved in conjugation are ATPases. Finding them in genomes and distinguishing them from other ATPases is challenging. Fourth, ICEs that are non-functional because of pseudogenization might be difficult to distinguish from functional elements.

In this work we present the results of a scan of prokaryotic genomes for conjugative systems in plasmids and chromosomes and the subsequent analysis to understand their functional and evolutionary relations. Previous studies provided precious insights of ICE evolution by analyzing closely related ICEs [38], [39]. Here we take the complementary approach and aim at the bigger picture. By searching for conjugative elements in all sequenced chromosomes and plasmids, we quantify the number of ICEs, characterize their diversity in terms of mechanism and phylogenetic representation, and study their evolution at the light of that of conjugative plasmids.

## Results

### Finding the C in ICE

If our assumption that ICEs and plasmids use similar conjugation machineries is correct we should be able to identify ICEs by using the sequence information of a large panel of proteins involved in plasmid conjugation. Previously, we carried out an analysis of plasmids by performing iterative similarity searches followed by protein clustering [14], but this approach poses problems of lack of convergence when using chromosomal data. Profile hidden Markov models (HMM) can retrieve more distant similarities than BLAST and do not pose as many problems of convergence as PSI-BLAST [40]. We therefore built protein profiles of the major representatives of the conjugation machinery using the information on proteins used in plasmid conjugation: relaxases (MOB types), T4CPs and VirB4s (see Materials and Methods). Additionally, we built profiles for proteins characteristic of each of the 4 types of T4SS found in plasmids of proteobacteria (see Materials and Methods). By using this approach, we did not need to use *ad hoc* methods to separate the ATPases (VirB4 and VirD4) because the hits of their profiles did not cross-match significantly. HMM protein profiles do not use the information of the new hits to change the protein profiles so they can be used reproductively upon change of the databank and independently of any reference dataset. We will soon make all the protein profiles available to the community by a web server. All the results of this scan are available in Dataset S1, including composition of all hits, accession numbers, gene names (with synonyms), and location in the replicons.

**Figure 2:**
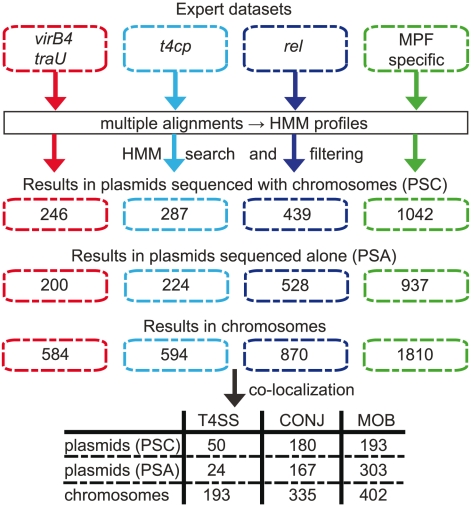
Methods and results of the identification pipeline. Upper. Diagram of the method used in the detection of the major representatives of the conjugation machinery: VirB4, T4CP, relaxases (rel) and the T4SS type-specific products. From expert datasets for the different proteins, we made multiple alignments and built HMM profiles that were used to scan chromosomes and plasmids. The numbers correspond to the number of hits. Lower. We then clustered co-localizing genes that are found within a maximum distance of 60 ORFs. A cluster containing a VirB4, a T4CP and a relaxase is considered as a putative conjugative system (CONJ). A cluster containing a VirB4 (plus or minus T4CP) but lacking a relaxase is considered as a putative protein-exporting T4SS. A cluster containing a relaxase but lacking a VirB4 is considered as a mobilizable element (MOB). The table shows the number of each type of clusters.

We scanned 3,489 replicons for the presence of conjugative systems, including 1,207 chromosomes, 891 plasmids sequenced along with chromosomes (PSC) and 1,391 plasmids that were sequenced alone, *i.e.* without the host chromosome(s) (PSA). Our analysis identified over 7000 proteins with significant matches (Figure 2). Close co-occurring hits were clustered together and this allowed the identification of putative T4SS. When a MOB and a T4CP neighbored a T4SS this locus was regarded as a conjugative system (see Materials and Methods). Conjugative loci in chromosomes were named ICEs. Our present results with plasmid sequences were very similar to those previously published [14] (see Methods). The comparison between chromosomes and the accompanying PSC plasmids allows an unbiased quantitative comparison between plasmids and ICEs in that both sets reflect the same sampling. Hence, we will show the results on all plasmids only when explicitly mentioned, otherwise all results concern the PSC plasmids. If we are correct in assuming homology between conjugative systems in ICEs and plasmids, we should be able to detect a large fraction of ICEs in prokaryotic genomes using information on proteins involved in plasmid conjugation. Indeed, we checked previously published lists of experimentally studied ICEs [20], [41] and were able to retrieve all for which experimental validation of mobility by self- conjugation and full sequence data were available (Table S1). Two mobilizable elements were missed in our analysis: Tn4555 [42] and NBUI1 [43]. These elements are mobilizable and have similar relaxases with no homolog in our genomic bank; as such, we did not include them in our study. We were thus able to identify all model ICEs in firmicutes (*e.g.* Tn916), bacteroides (*e.g.* CTnBST) and proteobacteria (*e.g.* SXT, ICEHin1056, ICEclc). The only exceptions were ICEs of actinobacteria that use FtsK-based transport systems within multi-cellular assemblages (e.g. pSAM2) [44], [45]. These systems transport dsDNA not ssDNA between cells within mycelia of some actinobacteria. As they don't contain relaxases neither T4SS these systems were not expected to be found in our analysis. Overall, these results indicate that using the accumulated body of knowledge on plasmid conjugation we can extensively identify and class ICEs.

### The prevalence of conjugative systems

Within the analyzed 1,124 complete prokaryotic genomes, which included the 1,207 chromosomes and their accompanying 891 PSC plasmids, we identified 335 putative ICEs and 180 putative conjugative plasmids. Additionally, we found 402 relaxases in chromosomes lacking neighboring T4SS. If these correspond to IMEs, the estimate of the ratio of conjugative over mobilizable elements both in chromosomes (ICE/IME = 0.83) as in PSC plasmids (ratio = 0.96) is approximately similar and lower than 1, suggesting that mobilization in *trans* is frequent in natural populations.

Naturally, mobilization in *trans* of an IME can only occur if the host genome encodes somewhere else a T4SS with the ability to build a compatible conjugative pilus. The frequency with which conjugative systems exist in prokaryotic cells is high. Overall, almost half of the genomes contain a T4SS, either in an ICE (18%), a conjugative plasmid (12%) or a T4SS without an accompanying relaxase (18%). Unfortunately, at this stage we cannot infer computationally if a given T4SS can mobilize another given mobilizable element in *trans*. Furthermore, we do not really know how often a T4SS is capable of mobilizing DNA in *trans*. Several T4SS that lack neighboring MOB and are involved in protein transport have this ability, *e.g.* the dot/icm system of *Legionella pneumophila* [46]. The *Bartonella tribocorum* T4SS can also complement deficiencies in the conjugative system of plasmid R388 [47], [48]. Further experimental work is required to assess the generality of these observations. An IME or mobilizable plasmid arriving at a cell has a probability of 30% of finding a conjugative element at the time of arrival. Naturally, given the high flux of these elements, if the mobilizable element remains long enough in the cell it will likely co-reside with a conjugative element.

**Figure 3:**
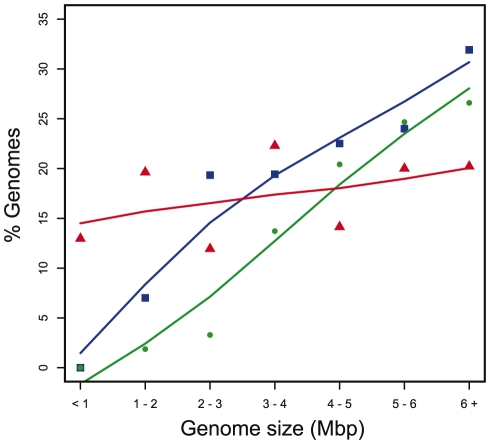
Percentage of genomes containing T4SS in function of genome size. Red: MOBless T4SS clusters T4SS+MOB-, Blue: ICE, Green: conjugative plasmids.

The probability that a cell harbors a conjugative element at a given moment depends on genome size (Figure 3). Small genomes rarely contain ICEs or conjugative plasmids, whereas large genomes often do so. This fits the common assumption that prokaryotes with smaller genomes engage more rarely in horizontal transfer. Nevertheless, several small genomes contain conjugative systems, as previously described for *Rickettsia* [49] and tenericutes [50]. Some T4SS have been present in the genomes of rickettsiales for a long period of time and their genomic organization is scattered, *i.e.* conjugation-related genes are not necessarily found in one single cluster [51]. We used the available literature to annotate these cases [51]. Analysis of the genomes of other proteobacteria suggests that this situation is relatively rare and that most conjugative systems are coded at one single cluster, which is required to ensure mobility of the locus upon transfer to a new recipient cell.

### ICEs are everywhere

**Figure 4:**
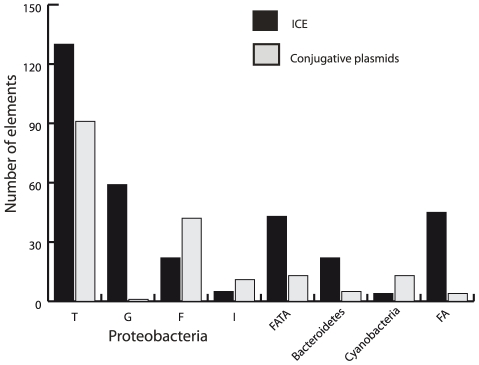
Distribution of ICEs and conjugative plasmids in the different groups of the VirB4 phylogenetic tree. (See also Figure 6.) The VirB4 families correspond to the four previously described proteobacterial MPF types (T, G, F and I) plus four additional families, associated with either host phyla (bacteroidetes, cyanobacteria) or mixtures of phyla (FA = firmicutes plus actinobacteria) or FATA (firmicutes, actinobacteria, tenericutes and archaea).

Using the method explained above we could make the first large-scale quantification of the abundance and diversity of ICEs among prokaryotes. We found ICEs in all bacterial clades where occurrences have been described previously, including the five major branches (α,β,γ,ε,δ) of proteobacteria, the bacteroidetes, and the firmicutes (Figure 4). In bacteroidetes, as well as in α- and β-proteobacteria, more than 50% of the available genomes contain at least one ICE. The other groups show relatively fewer ICEs, with these elements present in less than 30% of the genomes. We only found one ICE in archaea—in *Aciduliprofundum boonei*—and one conjugative PSC plasmid—plasmid pNG500 in *Haloarcula marismortui*. Yet, we found both in chromosomes and in plasmids many *bona fide* homologs of VirB4, often associated with a T4CP. It is possible that unknown relaxases exist in archaea, since conjugative plasmids are known in this clade and were included in our dataset [52], [53]. In actinobacteria, we found many MOB, but few T4SS or T4CP, both in plasmids and chromosomes. The rarity of T4SS in this clade could be explained by the alternative modes for DNA transfer within mycelia. Yet, elements in actinobacteria that are classed as mobilizable because they encode a relaxase presumably need a T4CP and T4SS to transfer as we know of no experimental evidence of functional interactions between relaxases and FtsK-based systems. Therefore, the number of conjugative systems in the clade still seems surprisingly low. Low sequence similarity is unlikely to be responsible for the lack of identifiable T4SS in actinobacteria since we can uncover distant homologs of VirB4 in all major clades of prokaryotes and we can even indentify by sequence similarity paralogous functionally unrelated ATPases. We found ICEs and conjugative plasmids in cyanobacteria. We had previously failed to do so [14], but the new protein profiles we built are more sensitive and show that this clade also contains conjugative systems both in plasmids and in chromosomes (to be published elsewhere). Additionally, we found ICEs in acidobacteria, in fusobacteria and one conjugative plasmid in chlorobi (pPAES01). In short, all clades with a significant number of sequenced genomes contain conjugative systems showing the ubiquity of this DNA transfer mechanism in the prokaryotic world.

### ICEs are more numerous than conjugative plasmids

While few ICEs have been experimentally studied in terms of conjugation, we found large numbers of them in the genomes of prokaryotes. Importantly, we found 86% more ICEs than conjugative plasmids (Figure 2 and Figure 3, p<0.001, binomial test). It should be emphasized that this is contrary to the expected if our method was biased, since we use information on plasmid conjugation systems to identify ICEs, not the other way around. Conjugative plasmids have been most thoroughly studied in proteobacteria whereas ICEs were discovered first in bacteroidetes and in firmicutes [54]. There is thus often a tendency to consider that conjugative plasmids are prevalent in proteobacteria and ICEs in the other two clades. Indeed, the preponderance of ICEs over conjugative plasmids varies between clades (Figure 4). In firmicutes and bacteroidetes ICEs do represent respectively 84% and 81% of all conjugative elements, while in proteobacteria ICEs only slightly outnumber conjugative plasmids. We identified no conjugative PSC plasmid within actinobacteria. Cyanobacteria were the only clade for which we found more conjugative plasmids (11) than ICEs (4). While we found conjugative plasmids in several different genera of cyanobacteria (*Cyanothece*, *Nostoc*, *Anabaena*, *Acaryochloris*), we only found ICEs in *Cyanothece*. Besides confirming the preconception that, in bacteroidetes and firmicutes, ICEs outnumber conjugative plasmids, we show that prevalence of ICEs over conjugative plasmids is almost general. ICEs might be more abundant in the analyzed genomes because of sequencing biases. First, certain sequencing projects might have ignored the sequencing of plasmids. Second, if ICEs are more stable in genomes than plasmids, bacterial culturing might induce a bias towards the over-representation of ICEs. In any case, our results clearly demonstrate that ICEs are a significant fraction of all conjugative elements in prokaryotes.

We next investigated if conjugation systems in plasmids and ICEs are of similar types. For this, we divided the conjugative systems found in proteobacteria into the four different archetypes: MPF_F_, MPF_T_, MPF_I_ and MPF_G_. MPF_T_ conjugative pili are short and thick, mate essentially in solid media and include elements such as CTn4371 [55] and MlSymR7A [56]. MPF_T_ are equally distributed, in relative terms, among conjugative plasmids and ICEs (Figure 4). Interestingly this is not the case for the other mating types that show significantly different frequencies among plasmids and ICEs (p<0.001, χ^2^ test). MPF_F_, which have long flexible pili, mate efficiently in solid and liquid, and include the SXT family [39]. These pili are rare among ICEs, whereas they are the second most frequent type in plasmids. On the other hand, the MPF_G_ pili have only been described to mate in solid surfaces [18] and are found essentially among ICEs, *e.g.* the clc or pKLC102 elements of *Pseudomonas* [57], [58]. We found few MPF_I_ systems in plasmids and even fewer in chromosomes. The latter were essentially found in the dot/icm systems of *Legionella* and *Coxiella*, where only the latter encode a MOB close to the T4SS. As a result, MPF types known to mate in liquid are under-represented in ICEs relative to plasmids.

### Co-occurrence of ICEs in genomes

We then analyzed the co-occurrence of ICEs in a given genome. Conjugative plasmids rarely code for two T4SS and, when they do, they tend to have multiple MPF_T_ [14]. We found 73 chromosomes encoding multiple ICEs and 32 genomes containing multiple conjugative plasmids. We found all MPF types in multiple copies, except for MPF_I_ in chromosomes and MPF_G_ in plasmids, but this could result from their rarity. A striking previously described case concerns *Orientia tsutsugamushi* genomes, which contain a large number of conjugation-related genes in clusters that for the most part present evidence of pseudogenization [59]. It is unclear in this case how many effective conjugation systems are encoded in the chromosome, but we could identify 5 complete clusters of MPF_F_. In our dataset the largest number of intact ICEs (seven) was found in *Bordetella petrii* DSM 12804 (which comprises both MPF_T_ and MPF_G_ elements) and in the firmicute *Clostridium difficile* 630. The genome of *Agrobacterium vitis* S4 contains the largest number of conjugative plasmids (4, all MPF_T_). In summary, conjugative systems in chromosomes and plasmids co-occur and sometimes in large numbers. This is expected, since each ICE is an independent element. This suggests that different types of T4SS can co-exist in a functional state in the cell. Discrimination between T4SS could be achieved by the specificity of the T4CP. Alternatively, one could imagine that in some cases conjugative elements also use T4SS encoded in *trans*.

### MOBless T4SS

One major surprising finding of this work was the high number of T4SS lacking nearby relaxases and thus not classed as ICEs (Figure 2). We can explain these findings in three different ways: as an artifact, as an indication of unknown relaxases or as evidence of high frequency of T4SS not involved in conjugation. Artifacts can occur in our analysis in several ways. First, one might have found many false positives in the detection of VirB4. This is unlikely because in proteobacteria (33% of MOBless T4SS), we find MOBless *virB4* genes neighboring other type-specific genes of T4SS (92 out of 109 clusters). This shows that in the vast majority of cases the *virB4* assignment in MOBless T4SS is correct. In the 17 remaining cases we almost always find at least one T4SS specific gene neighboring the MOBless *virB4* gene (16 out of 17 cases), but not enough to make it a valid cluster, suggesting that these loci correspond to inactive T4SS ongoing genetic degradation. Second, we might be failing to identify a large number of homologous T4CP or MOB in conjugative systems and this might lead to the misclassification of these clusters as MOBless T4SS. Yet, this does not fit the remaining observations: that MOBless T4SS are much more abundant in chromosomes than in plasmids and that we are able to identify VirB4, T4CP and MOB in clades distant from proteobacteria. All these pieces of evidence advocate against the hypothesis that the large number of MOBless T4SS is a consequence of methodological artifacts.

We showed above that the abundance of ICEs and conjugative plasmids depends strongly on genome size and that small genomes are practically devoid of conjugative systems (Figure 3). The distribution of MOBless T4SS is very different since these elements are abundant in small genomes and their frequency practically does not change with genome size (Figure 3). Small genomes tend to correspond to bacterial pathogens, and many of these are known to use T4SS to secrete proteins into the host cells for their subversion. T4SSs used for protein transport, as opposed to conjugation, have been described in strains of *Bartonella*, *Brucella*, *Bordetella*, the Legionellales, *Helicobacter*, and the Rickettsiales [46], [60]–[64]. Out of the 109 MOBless T4SS in proteobacteria, 77 are indeed found among these clades reinforcing the speculation that MOBless T4SS do often correspond to protein secretion systems. If so, this would include MPF_F_ elements, not known before to be recruited for that, and several clades of environmental prokaryotes, which so far were not known to carry such protein transport systems.

We have not yet done the precise delimitation of ICEs in genomes. Yet, we already carried out a preliminary analysis of the integrases co-localizing with the T4SSs to check for differences between ICEs and MOBless T4SSs. As described above, most ICEs include a tyrosine or serine recombinase and only a minority of well-characterized elements integrate by other means. Therefore the conjugation systems we identify in genomes are expected to have neighboring integrases. Co-localization of MOBless T4SS with integrases is expected under a number of situations: (i) if the protein secretion system is in a mobile element itself, as is frequently the case for T3SS [65], [66]; (ii) if it represents an element undergoing genetic degradation is which the relaxase was inactivated but not the integrase nor the T4SS genes; (iii) or if the genes encoding the T4SS happen to be near an unrelated mobile element. Yet, since integration is strictly necessary for ICE, we did expect to find more integrases neighboring the T4SS of ICE than those of MOBless T4SS. Using the PFAM domains (PF00589 for the tyrosine recombinases; PF07508 and PF00239 for serine recombinases), we found that within proteobacteria 87% of the ICEs and 50% of the MOBless T4SSs have a neighboring integrase distant no more than 60 genes from the conjugation-related genes. The difference is highly significant (p<0.001, binomial test) and suggests that MOBless T4SS are indeed intrinsically different from ICEs. We then analyzed the other clades to see if their MOBless T4SS were more frequently neighboring integrases since that could be the sign of the presence of unnoticed relaxases in these poorly studied genomes. We found that 90% of the ICEs and 56% of the MOBless T4SS in these other clades contain an integrase, within a distance of less than 60 genes, which is very close to the values found in proteobacteria. These results are consistent with intrinsic functional differences between the T4SS of ICEs and the MOBless T4SS.

**Figure 5:**
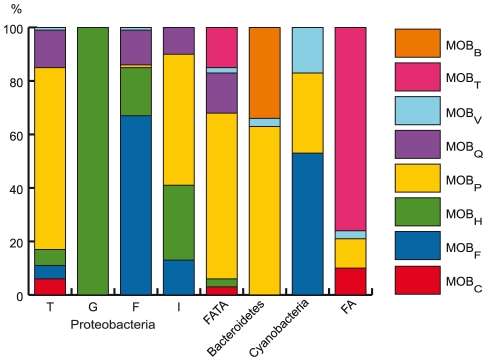
Distribution of the different MOB families among clades. (See also Figure 6.) The figure shows the percentage of each MOB type (color code at the right) associated to each MPF type.

Finally, we analyzed the co-occurrence of relaxases with T4SS in ICEs (Figure 5). Many MOB/MPF combinations are found among conjugative elements. This suggests that the MOB and MPF modules can shuffle over long evolutionary distances. However, there are some expected relevant associations between MPF and MOB, *e.g.* MPF_T_ with MOB_P_ or MPF_F_ with MOB_F_ as suggested by their frequent association in conjugative plasmids [14], [67]. Among less studied groups, MOB_B_ is specific of bacteroidetes and MPF_G_ only use one type of relaxase, MOB_H_ (58 cases in chromosomes and 2 in plasmids). It is therefore possible that some sub-types of T4SS use yet unknown relaxases. In particular, it is tempting to suggest that this is the case in archaea where we find very few relaxases.

### Evolutionary interplay between ICE and plasmids

**Figure 6:**
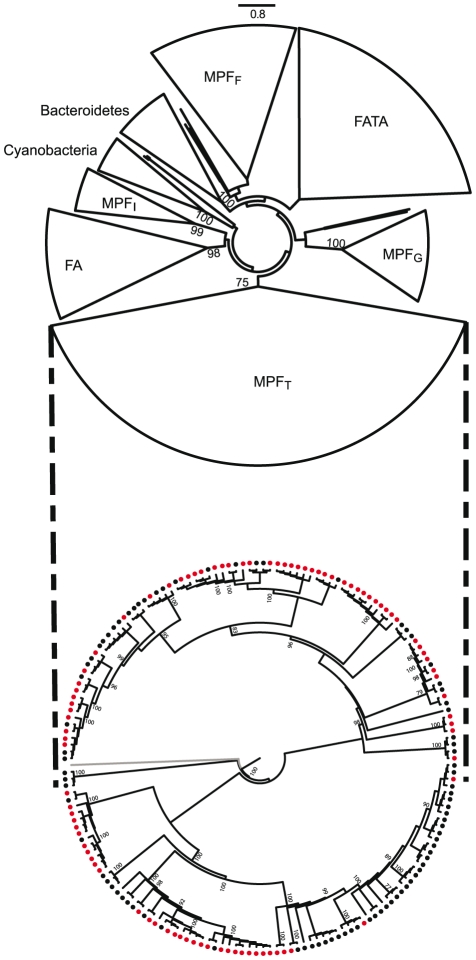
Phylogenetic analysis of a non-redundant subset of VirB4 proteins (only one protein per cluster of proteins >90% identical was used). Top: phylogeny of VirB4 with groups cartooned together. Bootstrap values above 75% are depicted. Bottom: phylogeny of VirB4 within MPF_T_ (in gray an outgroup to root the tree) with an indication if the gene is encoded in a chromosome (black circles) or in a plasmid (red).

As conjugation is an agent of horizontal transfer, and some very broad range plasmids have been described, one might expect little concordance between the phylogeny of VirB4 and that of the 16S rDNA. Yet, in plasmids it was found that large clades within bacteria corresponded to large clades in VirB4 with little apparent transfer between domains [14]. To check that similar results are still valid when using the information on ICEs and the new data on cyanobacteria and bacteroidetes, we made a phylogenetic analysis of the only ubiquitous element of T4SS: VirB4 (see Materials and Methods). This tree was built using a non-redundant subset of proteins and shows several remarkable things (Figure 6). First, MPF classification within proteobacteria remains meaningful, since the four types (F, G, I, T) are found in four monophyletic groups that exhibit strong support values. Both cyanobacteria and bacteroidetes form monophyletic clades, suggesting lack of significant transfer of conjugative systems between these and other clades since their divergence. This is consistent with their specific relaxases: MOB_V_ is mainly found in cyanobacteria and MOB_B_ is only found in bacteroidetes (Figure 5). Firmicutes and actinobacteria (FA in Figure 6), on one side, and firmicutes, actinobacteria, tenericutes and archaea (FATA in Figure 6), on the other, form the two remaining clades, but inside these groups one still finds mostly monophyletic clades. Thus, while elements propagating by means of conjugation systems are the most promiscuous known agents of horizontal transfer, the evolution of these systems does not show signs of frequent transfer of mobility backbone modules between types.

**Figure 7:**
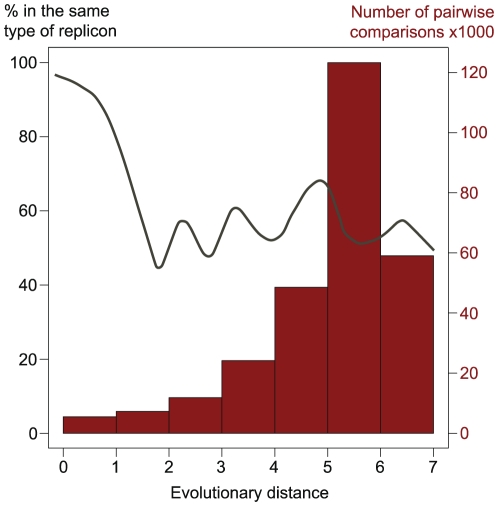
Analysis of phylogenetic associations between conjugative systems. The x-axis represents the pairwise evolutionary distances between VirB4 proteins (as taken from the tree in Figure 6). The histogram represents the counts in each bin of evolutionary distance (*e.g.* 5488 comparisons for proteins at an evolutionary distance less than 1). The line represents the frequency with which each of the two VirB4s at a given evolutionary distance belong to the same type of replicon, *i.e.* a plasmid or a chromosome. The line is a spline fit on the graph of pairwise evolutionary distances versus the frequency with each the two VirB4 are in the same type of replicon (*i.e.* both chromosomal or both plasmidic). For closely related sequences (smallest distances), the y-value is close to 100%, indicating that sequences belong to the same type of replicon. When the distance increases, the variable falls to 50%, indicating that the probability of finding a pair of VirB4 at these evolutionary distances in two chromosomes or in two plasmids is the same to that of finding them in a pair constituted by one ICE and one conjugative plasmid.

The existence of every type of T4SS in both chromosomes and plasmids of proteobacteria, albeit at very diverse frequencies, suggest that conjugative plasmids and ICEs have exchanged T4SS along their evolutionary history. To test this, we marked in the phylogenetic tree of VirB4 the respective genes that were encoded in chromosomes and in plasmids. An example for the MPF_T_ is presented in Figure 6. If ICEs were derived from conjugative plasmids, then one would expect large monophyletic clades of ICEs, indicating creation of the ICE, and clades devoid of ICEs, indicating lack of creation within the lineage. Furthermore, one would see evidence of plasmids as ancestral traits in the tree. If conjugative plasmids were derived from ICEs then the opposite picture should arise. The data presented in this work is not suggestive of any of these *scenarii*. Conjugative plasmids and ICEs (or chromosomal T4SS lacking nearby MOB) are intermingled along the whole tree (data not shown). At closer phylogenetic distances, *i.e.* the comparisons including the 15% of the tree closest to the tips, we do observe that the most similar VirB4 of an ICE is in general a VirB4 from another ICE and the reciprocal occurs for conjugative plasmids (Figure 7). We found 5 pairs of VirB4 encoded in different types of replicons that are distant by less than 1% in the tree. In three of the cases they are in a chromosome of one species and in a plasmid of another species within enterobacteria. Hence, at short evolutionary distances, plasmids and ICEs are indeed distinguishable. Yet, at slightly larger distances this signal quickly disappears and the ICEs and conjugative plasmids are perfectly mixed. The resulting picture is that one finds ICEs resembling much more some conjugative plasmids than other ICEs. For the most part of the evolutionary history of conjugation, ICEs have probably been converted to and from plasmids. As conjugative systems of both plasmids and ICEs shared most of their evolutionary history, they should be regarded as one and the same.

## Discussion

In this work we present the results of a semi-automatic method to detect conjugation-associated mobility systems not only in plasmids but also in chromosomes. This analysis paves the way for a systematic quantification of conjugation systems in prokaryotic genomes and in metagenomic data. When coupled with the detection of integration junctions (work in progress) it will also allow to analyze the gene repertoires of ICEs, and evaluate the evolutionary interplay between ICEs, conjugative plasmids and phages. Therefore, our present results only concern the C part of ICEs and conjugative plasmids. In the case of ICEs, this only gives an indication of their position in genomes, but not of their limits. ICEs can be very large (more than 500 kb for ICEMlSymR71 of *Mesorhizobium loti* [56]). Since the size of the C part is more or less constant, the variations in ICEs size will reveal the cargo genes they contain, much like for plasmids. The next step of this work will thus be to delimit ICEs within genomes in order to study the genes they carry. Our quantitative analysis shows that conjugative systems are more likely to be found in larger genomes. This fits the current assumption that larger genomes engage more frequently in horizontal gene transfer. The study of the cargo genes will help to quantify and qualify the role of ICEs in the functional diversification of prokaryotes.

Our analysis of MOBless T4SS in proteobacteria strongly suggests that many of these are involved in protein transport and not in conjugation. First, besides the archaeal clade, the relative frequency of these elements is similar in well-studied and poorly studied clades, suggesting this is not a methodological bias. Second, small genomes show abundant MOBless T4SS but practically no conjugative systems. This is in agreement with the utilization of MOBless T4SS in small genomes of pathogenic bacteria and in disagreement with the hypothesis that MOBless T4SSs are ICEs with unknown relaxases. Third, ICEs contain a significantly larger fraction of neighbouring integrases than MOBless T4SSs, both in proteobacteria and in the other clades. Fourth, a large fraction of the MOBless T4SSs in proteobacteria indeed corresponds to experimentally verified protein secretion systems or to orthologous systems in closely related genomes. If most MOBless T4SSs are indeed protein secretion systems, our results suggest that these systems are more frequent than previously suspected. Unexpectedly, many environmental bacteria have MOBless T4SSs, *e.g. Caulobacter*, *Thermoanaerobacter* or cyanobacteria. Protein secretion systems in these bacteria might be involved in antagonistic interactions with grazing protozoa, as was proposed for T3SS [68]. They could also be involved in protein transport, not associated with conjugation, or signaling interactions with other bacteria. To the best of our knowledge these functions have not yet been proposed for MOBless T4SSs. However, since conjugation is a form of protein secretion between prokaryotic cells, the evolution of a T4SS towards protein secretion between prokaryotes seems simpler than the evolution required to some of its other known functions, such as evolution into protein secretion into eukaryotic cells, or DNA uptake in *H. pylori*. Interestingly, *Agrobacterium tumefaciens vir* system exports both proteins and DNA at the time of conjugation of T-DNA into plants [69]. Protein secretion by MOBless T4SS might therefore be simple to evolve from a conjugative system.

We find that ICEs and conjugative plasmids use similar T4SS, but at different frequencies, especially concerning MPF_F_, which are more abundant in plasmids, and MPF_G_, which are present almost exclusively under the form of ICEs. The reasons for these preferences are puzzling and might be clarified by a better understanding of the conjugation mechanisms of Conjugation of ICEs is often assumed to take the same path as that of plasmids, once the element is excised and circularized. Differences in the two processes at the initial or final stages of conjugation might explain why one finds an enormous over-representation of ICEs in some clades whereas in cyanobacteria we find more conjugative plasmids.

Looking at the evolutionary relationships between ICEs and conjugative plasmids, we observed a close interplay between them in that the deepest clades in the VirB4 tree contain both types of elements. This strongly suggests that plasmids often become ICEs, and/or vice-versa. A plasmid might become an ICE upon acquisition of an integrase, e.g. from a phage, a genomic island or another ICE, although this is not strictly necessary, as documented in the Introduction. In fact, many plasmids contain some type of recombinase that could mediate site-specific integration or some type of DNA repeats that might allow integration by homologous recombination. Conversely, an ICE might become a plasmid upon acquisition of a REP system. Interestingly, some ICEs do contain REP systems (e.g. ICEBs1 [29]).

In conclusion, our results suggest that plasmids and ICEs might be just the two faces shown by a very similar type of element. One can speculate that plasmids disseminate to bacterial species in which they can replicate and to others in which they cannot. If the selection pressure for the presence of the element is high enough, the preservation of the element might be favored by its integration in the chromosome. This process can occur forwards and backwards so that we do not observe a terminal specialization between both types of element for some time. But certainly some ICEs end up stabilizing as chromosomal structures that remain as such for evolutionary long periods of time. What are the circumstances that drive them one way or another is a relevant question that remains to be answered.

## Materials and Methods

### Data

Data on complete prokaryotic chromosomes and plasmids was taken from Genbank Refseq (ftp://ftp.ncbi.nih.gov/genomes/Bacteria/). This data included 1,207 chromosomes, 891 plasmids associated with the chromosomes and 1,391 plasmids that were sequenced independently. We used the annotations of the Genbank files, removed all pseudogenes and all proteins with inner stop codons. The data on proteins of plasmid conjugation systems were taken from [14]. The following protein families were considered. **Relaxases** (see [67] for a description of each family, except MOB_B_, and MOB_T_): MOB_T_ (corresponding to protein Q47728 of *Enterococcus faecalis* conjugative transposon Tn916 Orf20 [70]), MOB_B_ (corresponding to mobilization protein B of *Bacteroides thetaiotaomicron* VPI-5482 [71]), MOB_V_, MOB_Q_, MOB_P_, MOB_H_, MOB_F_, MOB_C_. **Major ATPases**: VirB4 and TraU. **T4CP**: VirD4. **MPF_F_**: TraLEKVCWUcNHD. **MPF_T_**: VirB3689. **MPF_I_**: TraIKLMNPQRWY. **MPF_G_**: p31, p35, p41, p44, p51, p52.

### Construction of protein profiles

We took the data published previously [14], and for each protein family we repeated the analysis in that paper, *i.e.* we did PSI-BLAST of each key protein on chromosomes and plasmids and clustered the resulting proteins by MCL. This approach failed to produce good results because PSI-BLAST often did not converge in the searches made in chromosomes. For example, the searches for ATPases tend to put together many different ATPases of prokaryotes rendering their accurate separation difficult. We have thus used a different approach. For each protein family uncovered in our previous analysis of plasmids we did the following: (i) We carried out a multiple alignment with MUSCLE [72] and built a phylogenetic tree using PHYML [73]. With these two pieces of evidence we removed the very few cases of extreme divergence, the proteins that were too short and the proteins that were too long (typically false positives, fusions or fissions of proteins motivated by sequencing errors or pseudogenization). (ii) We built multiple alignments with MUSCLE of the selected proteins, checked manually the alignments and trimmed them to remove poorly aligned regions at the edges, if relevant. The C-terminal regions of MOB alignments were systematically trimmed, as suggested previously [67]. The alignment of the T4CP family showed two conserved regions separated by a region that aligned poorly. As a result, we split this alignment in two and made separate profiles with the two conserved regions. In general the two profiles were found together but only the second was found to be present in all conjugative elements apart some of those of the Tn916 family. These latter T4CP showed poor matches to the general T4CP profiles and we built one specific profile for this family. (iii) We used HMMER 3.0 to build protein profiles from the manually curated multiple alignments.

### Detection of elements of conjugative systems

We scanned the plasmid and chromosome sequences using the protein profiles and hmmsearch from HMMER 3.0 (http://hmmer.janelia.org/). Since this version of the program only does local alignment, we filtered the hits using a criterion of alignment size. In particular, we ignored all proteins that had a hit to the protein profile covering less than half of its length. Furthermore, we only kept for further analysis the proteins with at least one hit to the profile with a c-value <0.01. We then checked that the profiles matched significantly all the proteins in the protein families that originated the profile itself. Having thus obtained the hits of each gene in each replicon we analyzed them for cross-hits, *i.e.* proteins that matched significantly more than one profile. Some protein families with evidence of significant, albeit often weak, sequence similarity include VirB4 and TraU, VirB4 and T4CP and several of the MOB families. Proteins that hit significantly two families showed much better score to one family than to the other and we classed them using this information. A particular case concerns the hits between VirB3 and VirB4, since we often found these proteins as a fusion in one single peptide among MPF_T_. In this case we matched the two profiles and accounted the VirB4 profile for the possible presence of a T4SS and the VirB3 to its classification as an MPF_T_.

### Identification of loci implicated in conjugation

With the list of hits of each protein family we identified the putative conjugation loci. For this, we mapped the hits in replicons and clustered them together when they were encoded in the same region (less than 60 genes apart). Clusters of hits were defined transitively, *i.e.* they are successions of hits spaced by less than 60 genes. In practice, the clusters tend to be much smaller because the T4SS genes are coded in one or a few contiguous operons and the T4CP and the MOB also tend to be close. However, since ICE integrate from a circular form in chromosomes, the integration can lead to positioning of hits in opposite ends of the element, sometimes separating the MOB from the T4SS. We therefore checked by hand all occurrences of pairs of clusters that were between 60 and 100 genes apart. In the few cases where the clusters had complementary genes and where intervening genes did not correspond to prokaryotic housekeeping functions we put the clusters together. Protein export T4SS of *Rickettsiales* have been conserved for some time in these genomes and their genes have been scattered on the chromosome [51]. These clusters were reconstructed manually. We finally classified proteobacteria clusters using the 4 MPF types previously described [14]. A type is attributed to a cluster if the cluster contains at least 5, 4, 4, and 3 type-specific genes respectively for MPF_F_, MPF_G_, MPF_I_, MPF_T_.

### Tests

We made our initial analysis with HMMER 2.0 and then shifted to 3.0 because it is much faster. Yet, HMMER 3.0 only does local alignment and we tested if the HMMER 3.0 hits matching more than 50% of the domain were the same as the hits of the *glocal* approach in HMMER 2.0 (alignment local on the protein and global on the profile). We found that over 95% of the hits were retrieved by both approaches independently of the protein.

We then compared the results obtained on plasmids with this method and those from the previous study using PSI-BLAST+MCL [14]. Among the 250 conjugative plasmids that were previously identified, 241 have also been found by our new approach. There are some MOBs found by BLAST for which the HMMER local alignment was too short to pass our length criterion. The new procedure detected 97 conjugative plasmids that were missed by the previous one, *e.g.* due to the new hits among cyanobacteria that our previous approach missed.

### Phylogenetic inference

We made two types of phylogenetic analyses: (i) As a control for the presence of spurious elements in protein families. In this case we did maximum likelihood trees based on JTT model with PHYML [73]. (ii) To build the phylogenetic tree of VirB4. In this case to obtain a more accurate phylogeny we first aligned the proteins using MUSCLE [72] with default parameters as implemented in SeaView [74]. We removed all columns of the alignment containing more than 80% of gaps, and all the sequences that were more than 90% identical with another one in the alignment. We then tested the different protein models implemented in RAxML 7.2.7 [75] and chose the GTRGAMMA model since it gave the best likelihood. We built the tree by executing 100 replicates and keeping the best; we inferred 1000 bootstrap trees to obtain the confidence values of each node.

## Supporting Information

Dataset S1 Excel file with all identified clusters in three sheets: i) clusters in chromosomes; ii) clusters in plasmids sequenced with the chromosome; iii) other plasmids; iv–vi) all proteins with hits, genome position and synonyms. Brief description of the meaning of the different columns (with one example taken from first line of first sheet “Chrom”). 1) Accession number in GenBank (NC_008752), replicon (Acidovorax_avenae_subsp._citrulli_AAC00-1,_complete_genome._), clade (Proteobacteria), restricted clade (Betaproteobacteria), replicon type (circular), replicon size (5352772), number of genes in replicon (4709), presence or absence of T4SS (T4SS), conjugative/mobilizable/non-mobilizable(CONJ), MPF type (G), class including whether it contains T4SS, T4CP, relaxase and MPF type followed by an identification number (T4SS_T4CP_MOB_G_2), start and end of cluster in genome (in terms of genes) (478 and 556). This is followed by a list of columns with the hits for each profile. These hits are under the form *e.g.* virb4@ACAV001c01_004910&0.81018&3.4e-86, where virb4 is the profile for VirB4, followed by the gene name and the c-value of the hmmer hit. Some cells contain multiple entries corresponding to multiple hits. When the profile was not hit it only shows the profile name, *e.g.* MOBC. The MPF specific profiles are indicated in the form MPFtype_profile, *e.g.* F_traW for the TraW of MPF_F_ plasmid profile.
(XLS)

Table S1 List of experimentally studied ICEs and the results of our detection procedure on these elements.
(DOC)

## References

[1] Ochman H, Lawrence JG, Groisman EA (2000). Lateral gene transfer and the nature of bacterial innovation.. Nature.

[2] de la Cruz F, Davies J (2000). Horizontal gene transfer and the origin of species: lessons from bacteria.. Trends Microbiol.

[3] Tettelin H, Riley D, Cattuto C, Medini D (2008). Comparative genomics: the bacterial pan-genome.. Curr Opin Microbiol.

[4] Lorenz MG, Wackernagel W (1994). Bacterial gene transfer by natural genetic transformation in the environment.. Microbiol Rev.

[5] Brochet M, Rusniok C, Couve E, Dramsi S, Poyart C (2008). Shaping a bacterial genome by large chromosomal replacements, the evolutionary history of Streptococcus agalactiae.. Proc Natl Acad Sci U S A.

[6] Norman A, Hansen LH, Sorensen SJ (2009). Conjugative plasmids: vessels of the communal gene pool.. Philos Trans R Soc Lond B Biol Sci.

[7] Halary S, Leigh JW, Cheaib B, Lopez P, Bapteste E (2010). Network analyses structure genetic diversity in independent genetic worlds.. Proc Natl Acad Sci U S A.

[8] Kloesges T, Popa O, Martin W, Dagan T (2011). Networks of Gene Sharing among 329 Proteobacterial Genomes Reveal Differences in Lateral Gene Transfer Frequency at Different Phylogenetic Depths.. Mol Biol Evol.

[9] Amábile-Cuevas CF, Chicurel ME (1992). Bacterial plasmids and gene flux.. Cell.

[10] Sebaihia M, Wren BW, Mullany P, Fairweather NF, Minton N (2006). The multidrug-resistant human pathogen *Clostridium difficile* has a highly mobile, mosaic genome.. Nat Genet.

[11] Thomas CM (2000). Horizontal Gene Pool: Bacterial Plasmids and Gene Spread.

[12] van der Meer JR, Sentchilo V (2003). Genomic islands and the evolution of catabolic pathways in bacteria.. Curr Opin Biotechnol.

[13] de la Cruz F, Frost LS, Meyer RJ, Zechner E (2010). Conjugative DNA Metabolism in Gram-negative Bacteria.. FEMS Microbiol Rev.

[14] Smillie C, Garcillan-Barcia MP, Francia MV, Rocha EP, de la Cruz F (2010). Mobility of plasmids.. Microbiol Mol Biol Rev.

[15] Thompson DV, Melchers LS, Idler KB, Schilperoort RA, Hooykaas PJ (1988). Analysis of the complete nucleotide sequence of the Agrobacterium tumefaciens virB operon.. Nucleic Acids Res.

[16] Lawley TD, Klimke WA, Gubbins MJ, Frost LS (2003). F factor conjugation is a true type IV secretion system.. FEMS Microbiol Lett.

[17] Sampei G, Furuya N, Tachibana K, Saitou Y, Suzuki T (2010). Complete genome sequence of the incompatibility group I1 plasmid R64.. Plasmid.

[18] Juhas M, Crook DW, Dimopoulou ID, Lunter G, Harding RM (2007). Novel type IV secretion system involved in propagation of genomic islands.. J Bacteriol.

[19] Burrus V, Pavlovic G, Decaris B, Guedon G (2002). Conjugative transposons: the tip of the iceberg.. Mol Microbiol.

[20] Wozniak RA, Waldor MK (2010). Integrative and conjugative elements: mosaic mobile genetic elements enabling dynamic lateral gene flow.. Nat Rev Microbiol.

[21] Hochhut B, Waldor MK (1999). Site-specific integration of the conjugal *Vibrio cholerae* SXT element into prfC.. Mol Microbiol.

[22] Ravatn R, Studer S, Zehnder AJ, van der Meer JR (1998). Int-B13, an unusual site-specific recombinase of the bacteriophage P4 integrase family, is responsible for chromosomal insertion of the 105-kilobase clc element of *Pseudomonas* sp. Strain B13.. J Bacteriol.

[23] Caparon MG, Scott JR (1989). Excision and insertion of the conjugative transposon Tn916 involves a novel recombination mechanism.. Cell.

[24] Rajeev L, Malanowska K, Gardner JF (2009). Challenging a paradigm: the role of DNA homology in tyrosine recombinase reactions.. Microbiol Mol Biol Rev.

[25] Brochet M, Da Cunha V, Couve E, Rusniok C, Trieu-Cuot P (2009). Atypical association of DDE transposition with conjugation specifies a new family of mobile elements.. Mol Microbiol.

[26] Wang H, Mullany P (2000). The large resolvase TndX is required and sufficient for integration and excision of derivatives of the novel conjugative transposon Tn5397.. J Bacteriol.

[27] Chumley FG, Menzel R, Roth JR (1979). Hfr formation directed by tn10.. Genetics.

[28] Casey J, Daly C, Fitzgerald GF (1991). Chromosomal integration of plasmid DNA by homologous recombination in *Enterococcus faecalis* and *Lactococcus lactis* subsp. lactis hosts harboring Tn919.. Appl Environ Microbiol.

[29] Lee CA, Babic A, Grossman AD (2010). Autonomous plasmid-like replication of a conjugative transposon.. Mol Microbiol.

[30] Doublet B, Boyd D, Mulvey MR, Cloeckaert A (2005). The Salmonella genomic island 1 is an integrative mobilizable element.. Mol Microbiol.

[31] Douard G, Praud K, Cloeckaert A, Doublet B (2010). The Salmonella genomic island 1 is specifically mobilized in trans by the IncA/C multidrug resistance plasmid family.. PLoS ONE.

[32] Daccord A, Ceccarelli D, Burrus V (2010). Integrating conjugative elements of the SXT/R391 family trigger the excision and drive the mobilization of a new class of Vibrio genomic islands.. Mol Microbiol.

[33] Antonenka U, Nolting C, Heesemann J, Rakin A (2005). Horizontal transfer of Yersinia high-pathogenicity island by the conjugative RP4 attB target-presenting shuttle plasmid.. Mol Microbiol.

[34] Dobrindt U, Hochhut B, Hentschel U, Hacker J (2004). Genomic islands in pathogenic and environmental microorganisms.. Nat Rev Microbiol.

[35] Hofreuter D, Odenbreit S, Haas R (2001). Natural transformation competence in *Helicobacter pylori* is mediated by the basic components of a type IV secretion system.. Mol Microbiol.

[36] Llosa M, Roy C, Dehio C (2009). Bacterial type IV secretion systems in human disease.. Mol Microbiol.

[37] Frank AC, Alsmark CM, Thollesson M, Andersson SG (2005). Functional divergence and horizontal transfer of type IV secretion systems.. Mol Biol Evol.

[38] Juhas M, Power PM, Harding RM, Ferguson DJ, Dimopoulou ID (2007). Sequence and functional analyses of *Haemophilus* spp. genomic islands.. Genome Biol.

[39] Wozniak RA, Fouts DE, Spagnoletti M, Colombo MM, Ceccarelli D (2009). Comparative ICE genomics: insights into the evolution of the SXT/R391 family of ICEs.. PLoS Genet.

[40] Eddy SR (1998). Profile hidden Markov models.. Bioinformatics.

[41] Burrus V, Waldor MK (2004). Shaping bacterial genomes with integrative and conjugative elements.. Res Microbiol.

[42] Smith CJ, Parker AC (1993). Identification of a circular intermediate in the transfer and transposition of Tn4555, a mobilizable transposon from Bacteroides spp.. J Bacteriol.

[43] Shoemaker NB, Wang GR, Stevens AM, Salyers AA (1993). Excision, transfer, and integration of NBU1, a mobilizable site-selective insertion element.. J Bacteriol.

[44] Grohmann E, Muth G, Espinosa M (2003). Conjugative plasmid transfer in gram-positive bacteria.. Microbiol Mol Biol Rev.

[45] Vogelmann J, Ammelburg M, Finger C, Guezguez J, Linke D (2011). Conjugal plasmid transfer in *Streptomyces* resembles bacterial chromosome segregation by FtsK/SpoIIIE.. Embo J.

[46] Vogel J, Andrews H, Wong S, Isberg R (1998). Conjugative transfer by the virulence system of *Legionella pneumophila*.. Science.

[47] Seubert A, Hiestand R, de la Cruz F, Dehio C (2003). A bacterial conjugation machinery recruited for pathogenesis.. Mol Microbiol.

[48] de Paz HD, Sangari FJ, Bolland S, Garcia-Lobo JM, Dehio C (2005). Functional interactions between type IV secretion systems involved in DNA transfer and virulence.. Microbiology.

[49] Blanc G, Ogata H, Robert C, Audic S, Claverie JM (2007). Lateral gene transfer between obligate intracellular bacteria: evidence from the *Rickettsia massiliae* genome.. Genome Res.

[50] Marenda M, Barbe V, Gourgues G, Mangenot S, Sagne E (2006). A new integrative conjugative element occurs in *Mycoplasma agalactiae* as chromosomal and free circular forms.. J Bacteriol.

[51] Weinert L, Welch J, FM J (2009). Conjugation genes are common throughout the genus *Rickettsia* and are transmitted horizontally.. Proc Biol Sci.

[52] Prangishvili D, Albers S, Holz I, Arnold H, Stedman K (1998). Conjugation in archaea: frequent occurrence of conjugative plasmids in *Sulfolobus*.. Plasmid.

[53] Stedman KM, She Q, Phan H, Holz I, Singh H (2000). pING family of conjugative plasmids from the extremely thermophilic archaeon *Sulfolobus islandicus*: insights into recombination and conjugation in Crenarchaeota.. J Bacteriol.

[54] Salyers AA, Shoemaker NB, Stevens AM, Li LY (1995). Conjugative transposons: an unusual and diverse set of integrated gene transfer elements.. Microbiol Rev.

[55] Merlin C, Springael D, Toussaint A (1999). Tn4371: A modular structure encoding a phage-like integrase, a Pseudomonas-like catabolic pathway, and RP4/Ti-like transfer functions.. Plasmid.

[56] Sullivan JT, Ronson CW (1998). Evolution of rhizobia by acquisition of a 500-kb symbiosis island that integrates into a phe-tRNA gene.. Proc Natl Acad Sci U S A.

[57] Klockgether J, Reva O, Larbig K, Tummler B (2004). Sequence analysis of the mobile genome island pKLC102 of *Pseudomonas aeruginosa* C.. J Bacteriol.

[58] Gaillard M, Vallaeys T, Vorholter FJ, Minoia M, Werlen C (2006). The clc element of *Pseudomonas* sp. strain B13, a genomic island with various catabolic properties.. J Bacteriol.

[59] Cho NH, Kim HR, Lee JH, Kim SY, Kim J (2007). The *Orientia tsutsugamushi* genome reveals massive proliferation of conjugative type IV secretion system and host-cell interaction genes.. Proc Natl Acad Sci U S A.

[60] Parkhill J, Sebaihia M, Preston A, Murphy LD, Thomson N (2003). Comparative analysis of the genome sequences of *Bordetella pertussis*, *Bordetella parapertussis* and *Bordetella bronchiseptica*.. Nat Genet.

[61] Alvarez-Martinez CE, Christie PJ (2009). Biological diversity of prokaryotic type IV secretion systems.. Microbiol Mol Biol Rev.

[62] Engel P, Salzburger W, Liesch M, Chang CC, Maruyama S (2011). Parallel Evolution of a Type IV Secretion System in Radiating Lineages of the Host-Restricted Bacterial Pathogen Bartonella.. PLoS Genet.

[63] Fischer W, Windhager L, Rohrer S, Zeiller M, Karnholz A (2010). Strain-specific genes of *Helicobacter pylori*: genome evolution driven by a novel type IV secretion system and genomic island transfer.. Nucleic Acids Res.

[64] Nystedt B, Frank AC, Thollesson M, Andersson SG (2008). Diversifying selection and concerted evolution of a type IV secretion system in Bartonella.. Mol Biol Evol.

[65] Medini D, Covacci A, Donati C (2006). Protein homology network families reveal step-wise diversification of Type III and Type IV secretion systems.. PLoS Comput Biol.

[66] Naum M, Brown EW, Mason-Gamer RJ (2009). Phylogenetic evidence for extensive horizontal gene transfer of type III secretion system genes among enterobacterial plant pathogens.. Microbiology.

[67] Garcillan-Barcia MP, Francia MV, de la Cruz F (2009). The diversity of conjugative relaxases and its application in plasmid classification.. FEMS Microbiol Rev.

[68] Preston GM (2007). Metropolitan microbes: type III secretion in multihost symbionts.. Cell Host Microbe.

[69] Lacroix B, Tzfira T, Vainstein A, Citovsky V (2006). A case of promiscuity: Agrobacterium's endless hunt for new partners.. Trends Genet.

[70] Rocco JM, Churchward G (2006). The integrase of the conjugative transposon Tn916 directs strand- and sequence-specific cleavage of the origin of conjugal transfer, oriT, by the endonuclease Orf20.. J Bacteriol.

[71] Xu J, Bjursell MK, Himrod J, Deng S, Carmichael LK (2003). A genomic view of the human-Bacteroides thetaiotaomicron symbiosis.. Science.

[72] Edgar RC (2004). MUSCLE: multiple sequence alignment with high accuracy and high throughput.. Nucleic Acids Res.

[73] Anisimova M, Gascuel O (2006). Approximate likelihood-ratio test for branches: A fast, accurate, and powerful alternative.. Syst Biol.

[74] Gouy M, Guindon S, Gascuel O (2010). SeaView version 4: A multiplatform graphical user interface for sequence alignment and phylogenetic tree building.. Mol Biol Evol.

[75] Stamatakis A (2006). RAxML-VI-HPC: maximum likelihood-based phylogenetic analyses with thousands of taxa and mixed models.. Bioinformatics.

