# Supplementary material for: The Repertoire of ICE in Prokaryotes Underscores the Unity, Diversity, and Ubiquity of Conjugation
Source: PLoS Genet. 2011 Aug 18;7(8):e1002222. doi: 10.1371/journal.pgen.1002222 (PMC3158045; doi:10.1371/journal.pgen.1002222)
Supplement: Table S1 — List of experimentally studied ICEs and the results of our detection procedure on these elements. (DOC) [file pgen.1002222.s002.doc]

| Supplementary table 1 – List of experimentally studied ICEs and the results of our detection procedure on these elements. | | | | |
| --- | --- | --- | --- | --- |
| **ICE/IME** | **Host** | **GenBank Accesion number** | **Experimental evidence** | **Detected?** |
| SXT | *Vibrio cholerae* O1 biovar El Tor | AB450045.1 | Excision [1], conjugation [2-5], and integration [6, 1, 7, 8] | Yes MPF_F_ |
| R391 | *Providencia rettgeri* | AY090559.1 | Excision [9], conjugation [2, 3], and integration [7, 10] | Yes MPF_F_ |
| ICE*Pda*Spa1 | *Photobacterium damselae* subsp. piscida | AJ870986.2 | Excision [11], conjugation [11], integration [11] | Yes MPF_F_ |
| ICE*Bs*1 | *Bacillus subtilis* | AB001488.1 | Excision [13-17], conjugation [18, 19, 14, 20, 21], and integration [17] | Yes |
| PAPI-1 | *Pseudomonas aeruginosa* PA14 | AY273869.1 | Excision [22], conjugation [23, 22], and integration [22] | Yes MPF_G_ |
| ICE*clc* | *Pseudomonas* sp. strain B13 | AJ617740.1 | Excision [24-26], conjugation [24, 27, 28], and integration [29, 30, 26, 31] | Yes MPF_G_ |
| GI3 | *Bordetella petrii* strain DSM 12804 | NC_010170.1 *(coordinates 1493558-1595651)* | Excision [32], conjugation [32], and integration [32] | Yes MPF_G_ |
| ICE*MlSymR7A* | *Mesorhizobium loti* strain R7A | AL672112.1  AL672113.1  AL672114.1  AL672115.1 | Excision [33, 34], conjugation [33-35], and integration [34, 35] | Yes MPF_T_ |
| ICE*Hin1056* | *Haemophilus influenzae* | AJ627386.1 | Conjugation [36], and integration [37] | Yes MPF_G_ |
| Tn*916* | *Enterococcus faecalis* | NC_006372.1 | Excision [38-42], conjugation [43-46], and integration [43, 47, 46, 42] | Yes |
| Tn*GBS2* | *Streptococcus agalactiae* NEM316 | AL766848.1  AL766849.1  (from gbs1118 to gbs1153) | Excision [54], conjugation [54], and integration [54] | Yes |
| RD2 | *Streptococcus pyogenes* strain MGAS6180 | CP000056.1  (from M28_Spy1303 to M28_Spy1337) | Excision [55], conjugation [55], and integration [55] | Yes |
| CTn*341* | *Bacteroides fragilis* | AY515263.1 | Excision [48], conjugation [56], and integration [48] | Yes |
| ICE*Ec*1 | *Escherichia coli* strain ECOR31 | AY233333.1 | Excision [57] | Yes MPF_T_ |
| pKLC102 | *Pseudomonas aeruginosa* strain C | AY257538.1 | Excision [58] | Yes MPF_G_ |
| ICE*Ec*2 | *Escherichia coli* strain BEN374 | GU725392.1 | Excision [59], conjugation [59], and integration [59] | Yes MPF_G_ |
| ICE*Kp*1 | *Klebsiella pneumoniae* | AB298504.1 | Excision [60, 61], conjugation [60] and integration [60, 61] | Yes MPF_T_ |
| ICE*St1* | *Streptococcus thermophilus* | AJ278471.1 | Excision [62, 63], conjugation [64], and integration [64] | Yes |
| ICE*St3* | *Streptococcus thermophilus* | AJ586568.1 | Excision [62, 65], conjugation [64], and integration [64] | Yes |
| Tn*4555* | *Bacteroides fragilis* | U75371.3 | Excision [70] [71], mobilization [71, 72] integration [73-75] | No Missing relaxase |
| CTn*BST* | *Bacteroides uniformis* | AY345595.2 | Excision [68], conjugation [69, 68], and integration [68] | Yes |
| Tn*1549* | *Enterococcus faecalis* | AF192329.1 | Mobilization [76, 77]  Integration [76] | Yes |
| Tn*4371* | *Ralstonia oxalatica* | AJ536756 | Excision [79], mobilization [79, 80], integration [79] | Yes MPF_T_ |
| NBU1 (IME) | *Bacteroides uniformis* | NC_006373.1 | Excision [83], mobilization [84, 83], integration [49, 85-87, 83] | No Missing relaxase |
| ICE*6013* | *Staphylococcus aureus* strain HDG2 | FJ231270.1 | Excision [88] | Yes |
| ICE*Sde3396* | *Streptococcus dysgalactiae* subsp. equisimilis strain NS3396 | EU142041.1 | Conjugation [89] | Yes |

1. Burrus V, Waldor MK (2003) Control of SXT integration and excision*.* Journal of bacteriology 185: 5045-5054.

2. Ceccarelli D, Daccord A, Rene M, Burrus V (2008) Identification of the origin of transfer (oriT) and a new gene required for mobilization of the SXT/R391 family of integrating conjugative elements*.* Journal of bacteriology 190: 5328-5338.

3. Marrero J, Waldor MK (2007) Determinants of entry exclusion within Eex and TraG are cytoplasmic*.* Journal of bacteriology 189: 6469-6473.

4. McLeod SM, Burrus V, Waldor MK (2006) Requirement for Vibrio cholerae integration host factor in conjugative DNA transfer*.* Journal of bacteriology 188: 5704-5711.

5. Waldor MK, Tschape H, Mekalanos JJ (1996) A new type of conjugative transposon encodes resistance to sulfamethoxazole, trimethoprim, and streptomycin in Vibrio cholerae O139*.* Journal of bacteriology 178: 4157-4165.

6. Beaber JW, Hochhut B, Waldor MK (2002) Genomic and functional analyses of SXT, an integrating antibiotic resistance gene transfer element derived from Vibrio cholerae*.* Journal of bacteriology 184: 4259-4269.

7. Hochhut B, Beaber JW, Woodgate R, Waldor MK (2001) Formation of chromosomal tandem arrays of the SXT element and R391, two conjugative chromosomally integrating elements that share an attachment site*.* Journal of bacteriology 183: 1124-1132.

8. Hochhut B, Waldor MK (1999) Site-specific integration of the conjugal Vibrio cholerae SXT element into prfC*.* Molecular microbiology 32: 99-110.

9. O'Halloran JA, McGrath BM, Pembroke JT (2007) The orf4 gene of the enterobacterial ICE, R391, encodes a novel UV-inducible recombination directionality factor, Jef, involved in excision and transfer of the ICE*.* FEMS microbiology letters 272: 99-105.

10. McGrath BM, Pembroke JT (2004) Detailed analysis of the insertion site of the mobile elements R997, pMERPH, R392, R705 and R391 in E. coli K12*.* FEMS microbiology letters 237: 19-26.

11. Osorio CR, Marrero J, Wozniak RA, Lemos ML, Burrus V, et al. (2008) Genomic and functional analysis of ICEPdaSpa1, a fish-pathogen-derived SXT-related integrating conjugative element that can mobilize a virulence plasmid*.* Journal of bacteriology 190: 3353-3361.

12. Harada S, Ishii Y, Saga T, Tateda K, Yamaguchi K (2010) Chromosomally encoded blaCMY-2 located on a novel SXT/R391-related integrating conjugative element in a Proteus mirabilis clinical isolate*.* Antimicrobial agents and chemotherapy 54: 3545-3550.

13. Auchtung JM, Lee CA, Garrison KL, Grossman AD (2007) Identification and characterization of the immunity repressor (ImmR) that controls the mobile genetic element ICEBs1 of Bacillus subtilis*.* Molecular microbiology 64: 1515-1528.

14. Berkmen MB, Lee CA, Loveday EK, Grossman AD (2010) Polar positioning of a conjugation protein from the integrative and conjugative element ICEBs1 of Bacillus subtilis*.* Journal of bacteriology 192: 38-45.

15. Bose B, Grossman AD (2011) Regulation of horizontal gene transfer in Bacillus subtilis by activation of a conserved site-specific protease*.* Journal of bacteriology 193: 22-29.

16. Goranov AI, Kuester-Schoeck E, Wang JD, Grossman AD (2006) Characterization of the global transcriptional responses to different types of DNA damage and disruption of replication in Bacillus subtilis*.* Journal of bacteriology 188: 5595-5605.

17. Lee CA, Auchtung JM, Monson RE, Grossman AD (2007) Identification and characterization of int (integrase), xis (excisionase) and chromosomal attachment sites of the integrative and conjugative element ICEBs1 of Bacillus subtilis*.* Molecular microbiology 66: 1356-1369.

18. Auchtung JM, Lee CA, Monson RE, Lehman AP, Grossman AD (2005) Regulation of a Bacillus subtilis mobile genetic element by intercellular signaling and the global DNA damage response*.* Proceedings of the National Academy of Sciences of the United States of America 102: 12554-12559.

19. Babic A, Berkmen MB, Lee CA, Grossman AD (2011) Efficient gene transfer in bacterial cell chains*.* mBio 2:

20. Grohmann E Conjugative transfer of the integrative and conjugative element ICEBs1 from Bacillus subtilis likely initiates at the donor cell pole*.* Journal of bacteriology 192: 23-25.

21. Lee CA, Grossman AD (2007) Identification of the origin of transfer (oriT) and DNA relaxase required for conjugation of the integrative and conjugative element ICEBs1 of Bacillus subtilis*.* Journal of bacteriology 189: 7254-7261.

22. Qiu X, Gurkar AU, Lory S (2006) Interstrain transfer of the large pathogenicity island (PAPI-1) of Pseudomonas aeruginosa*.* Proceedings of the National Academy of Sciences of the United States of America 103: 19830-19835.

23. Carter MQ, Chen J, Lory S (2010) The Pseudomonas aeruginosa pathogenicity island PAPI-1 is transferred via a novel type IV pilus*.* Journal of bacteriology 192: 3249-3258.

24. Gaillard M, Pernet N, Vogne C, Hagenbuchle O, van der Meer JR (2008) Host and invader impact of transfer of the clc genomic island into Pseudomonas aeruginosa PAO1*.* Proceedings of the National Academy of Sciences of the United States of America 105: 7058-7063.

25. Minoia M, Gaillard M, Reinhard F, Stojanov M, Sentchilo V, et al. (2008) Stochasticity and bistability in horizontal transfer control of a genomic island in Pseudomonas*.* Proceedings of the National Academy of Sciences of the United States of America 105: 20792-20797.

26. Sentchilo V, Czechowska K, Pradervand N, Minoia M, Miyazaki R, et al. (2009) Intracellular excision and reintegration dynamics of the ICEclc genomic island of Pseudomonas knackmussii sp. strain B13*.* Molecular microbiology 72: 1293-1306.

27. Miyazaki R, van der Meer JR A dual functional origin of transfer in the ICEclc genomic island of Pseudomonas knackmussii B13*.* Molecular microbiology 79: 743-758.

28. Ravatn R, Zehnder AJ, van der Meer JR (1998) Low-frequency horizontal transfer of an element containing the chlorocatechol degradation genes from Pseudomonas sp. strain B13 to Pseudomonas putida F1 and to indigenous bacteria in laboratory-scale activated-sludge microcosms*.* Applied and environmental microbiology 64: 2126-2132.

29. Ravatn R, Studer S, Springael D, Zehnder AJ, van der Meer JR (1998) Chromosomal integration, tandem amplification, and deamplification in Pseudomonas putida F1 of a 105-kilobase genetic element containing the chlorocatechol degradative genes from Pseudomonas sp. Strain B13*.* Journal of bacteriology 180: 4360-4369.

30. Ravatn R, Studer S, Zehnder AJ, van der Meer JR (1998) Int-B13, an unusual site-specific recombinase of the bacteriophage P4 integrase family, is responsible for chromosomal insertion of the 105-kilobase clc element of Pseudomonas sp. Strain B13*.* Journal of bacteriology 180: 5505-5514.

31. Sentchilo V, Ravatn R, Werlen C, Zehnder AJ, van der Meer JR (2003) Unusual integrase gene expression on the clc genomic island in Pseudomonas sp. strain B13*.* Journal of bacteriology 185: 4530-4538.

32. Lechner M, Schmitt K, Bauer S, Hot D, Hubans C, et al. (2009) Genomic island excisions in Bordetella petrii*.* BMC microbiology 9: 141.

33. Ramsay JP, Sullivan JT, Jambari N, Ortori CA, Heeb S, et al. (2009) A LuxRI-family regulatory system controls excision and transfer of the Mesorhizobium loti strain R7A symbiosis island by activating expression of two conserved hypothetical genes*.* Molecular microbiology 73: 1141-1155.

34. Ramsay JP, Sullivan JT, Stuart GS, Lamont IL, Ronson CW (2006) Excision and transfer of the Mesorhizobium loti R7A symbiosis island requires an integrase IntS, a novel recombination directionality factor RdfS, and a putative relaxase RlxS*.* Molecular microbiology 62: 723-734.

35. Sullivan JT, Ronson CW (1998) Evolution of rhizobia by acquisition of a 500-kb symbiosis island that integrates into a phe-tRNA gene*.* Proceedings of the National Academy of Sciences of the United States of America 95: 5145-5149.

36. Juhas M, Power PM, Harding RM, Ferguson DJ, Dimopoulou ID, et al. (2007) Sequence and functional analyses of Haemophilus spp. genomic islands*.* Genome biology 8: R237.

37. Dimopoulou ID, Russell JE, Mohd-Zain Z, Herbert R, Crook DW (2002) Site-specific recombination with the chromosomal tRNA(Leu) gene by the large conjugative Haemophilus resistance plasmid*.* Antimicrobial agents and chemotherapy 46: 1602-1603.

38. Celli J, Poyart C, Trieu-Cuot P (1997) Use of an excision reporter plasmid to study the intracellular mobility of the conjugative transposon Tn916 in gram-positive bacteria*.* Microbiology (Reading, England) 143 ( Pt 4): 1253-1261.

39. Hinerfeld D, Churchward G (2001) Xis protein of the conjugative transposon Tn916 plays dual opposing roles in transposon excision*.* Molecular microbiology 41: 1459-1467.

40. Marra D, Pethel B, Churchward GG, Scott JR (1999) The frequency of conjugative transposition of Tn916 is not determined by the frequency of excision*.* Journal of bacteriology 181: 5414-5418.

41. Marra D, Scott JR (1999) Regulation of excision of the conjugative transposon Tn916*.* Molecular microbiology 31: 609-621.

42. Storrs MJ, Poyart-Salmeron C, Trieu-Cuot P, Courvalin P (1991) Conjugative transposition of Tn916 requires the excisive and integrative activities of the transposon-encoded integrase*.* Journal of bacteriology 173: 4347-4352.

43. Franke AE, Clewell DB (1981) Evidence for a chromosome-borne resistance transposon (Tn916) in Streptococcus faecalis that is capable of "conjugal" transfer in the absence of a conjugative plasmid*.* Journal of bacteriology 145: 494-502.

44. Jaworski DD, Clewell DB (1995) A functional origin of transfer (oriT) on the conjugative transposon Tn916*.* Journal of bacteriology 177: 6644-6651.

45. Rocco JM, Churchward G (2006) The integrase of the conjugative transposon Tn916 directs strand- and sequence-specific cleavage of the origin of conjugal transfer, oriT, by the endonuclease Orf20*.* Journal of bacteriology 188: 2207-2213.

46. Scott JR, Bringel F, Marra D, Van Alstine G, Rudy CK (1994) Conjugative transposition of Tn916: preferred targets and evidence for conjugative transfer of a single strand and for a double-stranded circular intermediate*.* Molecular microbiology 11: 1099-1108.

47. Lu F, Churchward G (1995) Tn916 target DNA sequences bind the C-terminal domain of integrase protein with different affinities that correlate with transposon insertion frequency*.* Journal of bacteriology 177: 1938-1946.

48. Bedzyk LA, Shoemaker NB, Young KE, Salyers AA (1992) Insertion and excision of Bacteroides conjugative chromosomal elements*.* Journal of bacteriology 174: 166-172.

49. Cheng Q, Paszkiet BJ, Shoemaker NB, Gardner JF, Salyers AA (2000) Integration and excision of a Bacteroides conjugative transposon, CTnDOT*.* Journal of bacteriology 182: 4035-4043.

50. Cheng Q, Sutanto Y, Shoemaker NB, Gardner JF, Salyers AA (2001) Identification of genes required for excision of CTnDOT, a Bacteroides conjugative transposon*.* Molecular microbiology 41: 625-632.

51. Sutanto Y, Shoemaker NB, Gardner JF, Salyers AA (2002) Characterization of Exc, a novel protein required for the excision of Bacteroides conjugative transposon*.* Molecular microbiology 46: 1239-1246.

52. Wang Y, Shoemaker NB, Salyers AA (2004) Regulation of a Bacteroides operon that controls excision and transfer of the conjugative transposon CTnDOT*.* Journal of bacteriology 186: 2548-2557.

53. Malanowska K, Yoneji S, Salyers AA, Gardner JF (2007) CTnDOT integrase performs ordered homology-dependent and homology-independent strand exchanges*.* Nucleic acids research 35: 5861-5873.

54. Brochet M, Da Cunha V, Couve E, Rusniok C, Trieu-Cuot P, et al. (2009) Atypical association of DDE transposition with conjugation specifies a new family of mobile elements*.* Molecular microbiology 71: 948-959.

55. Sitkiewicz I, Green NM, Guo N, Mereghetti L, Musser JM (2011) Lateral gene transfer of streptococcal ICE element RD2 (region of difference 2) encoding secreted proteins*.* BMC microbiology 11: 65.

56. Peed L, Parker AC, Smith CJ (2010) Genetic and functional analyses of the mob operon on conjugative transposon CTn341 from Bacteroides spp*.* Journal of bacteriology 192: 4643-4650.

57. Schubert S, Dufke S, Sorsa J, Heesemann J (2004) A novel integrative and conjugative element (ICE) of Escherichia coli: the putative progenitor of the Yersinia high-pathogenicity island*.* Molecular microbiology 51: 837-848.

58. Klockgether J, Wurdemann D, Reva O, Wiehlmann L, Tummler B (2007) Diversity of the abundant pKLC102/PAGI-2 family of genomic islands in Pseudomonas aeruginosa*.* Journal of bacteriology 189: 2443-2459.

59. Roche D, Flechard M, Lallier N, Reperant M, Bree A, et al. (2010) ICEEc2, a new integrative and conjugative element belonging to the pKLC102/PAGI-2 family, identified in Escherichia coli strain BEN374*.* Journal of bacteriology 192: 5026-5036.

60. Lin TL, Lee CZ, Hsieh PF, Tsai SF, Wang JT (2008) Characterization of integrative and conjugative element ICEKp1-associated genomic heterogeneity in a Klebsiella pneumoniae strain isolated from a primary liver abscess*.* Journal of bacteriology 190: 515-526.

61. Rakin A, Noelting C, Schropp P, Heesemann J (2001) Integrative module of the high-pathogenicity island of Yersinia*.* Molecular microbiology 39: 407-415.

62. Bellanger X, Morel C, Decaris B, Guedon G (2007) Derepression of excision of integrative and potentially conjugative elements from Streptococcus thermophilus by DNA damage response: implication of a cI-related repressor*.* Journal of bacteriology 189: 1478-1481.

63. Burrus V, Roussel Y, Decaris B, Guedon G (2000) Characterization of a novel integrative element, ICESt1, in the lactic acid bacterium Streptococcus thermophilus*.* Applied and environmental microbiology 66: 1749-1753.

64. Bellanger X, Roberts AP, Morel C, Choulet F, Pavlovic G, et al. (2009) Conjugative transfer of the integrative conjugative elements ICESt1 and ICESt3 from Streptococcus thermophilus*.* Journal of bacteriology 191: 2764-2775.

65. Pavlovic G, Burrus V, Gintz B, Decaris B, Guedon G (2004) Evolution of genomic islands by deletion and tandem accretion by site-specific recombination: ICESt1-related elements from Streptococcus thermophilus*.* Microbiology (Reading, England) 150: 759-774.

66. Doublet B, Boyd D, Mulvey MR, Cloeckaert A (2005) The Salmonella genomic island 1 is an integrative mobilizable element*.* Molecular microbiology 55: 1911-1924.

67. Douard G, Praud K, Cloeckaert A, Doublet B (2011) The Salmonella genomic island 1 is specifically mobilized in trans by the IncA/C multidrug resistance plasmid family*.* PloS one 5: e15302.

68. Wesslund NA, Wang GR, Song B, Shoemaker NB, Salyers AA (2007) Integration and excision of a newly discovered bacteroides conjugative transposon, CTnBST*.* Journal of bacteriology 189: 1072-1082.

69. Gupta A, Vlamakis H, Shoemaker N, Salyers AA (2003) A new Bacteroides conjugative transposon that carries an ermB gene*.* Applied and environmental microbiology 69: 6455-6463.

70. Parker AC, Smith CJ (2004) A multicomponent system is required for tetracycline-induced excision of Tn4555*.* Journal of bacteriology 186: 438-444.

71. Smith CJ, Parker AC (1993) Identification of a circular intermediate in the transfer and transposition of Tn4555, a mobilizable transposon from Bacteroides spp*.* Journal of bacteriology 175: 2682-2691.

72. Smith CJ, Parker AC (1998) The transfer origin for Bacteroides mobilizable transposon Tn4555 is related to a plasmid family from gram-positive bacteria*.* Journal of bacteriology 180: 435-439.

73. Bacic MK, Smith CJ (2005) Analysis of chromosomal insertion sites for Bacteroides Tn4555 and the role of TnpA*.* Gene 353: 80-88.

74. Tribble GD, Parker AC, Smith CJ (1997) The Bacteroides mobilizable transposon Tn4555 integrates by a site-specific recombination mechanism similar to that of the gram-positive bacterial element Tn916*.* Journal of bacteriology 179: 2731-2739.

75. Tribble GD, Parker AC, Smith CJ (1999) Transposition genes of the Bacteroides mobilizable transposon Tn4555: role of a novel targeting gene*.* Molecular microbiology 34: 385-394.

76. Launay A, Ballard SA, Johnson PD, Grayson ML, Lambert T (2006) Transfer of vancomycin resistance transposon Tn1549 from Clostridium symbiosum to Enterococcus spp. in the gut of gnotobiotic mice*.* Antimicrobial agents and chemotherapy 50: 1054-1062.

77. Tsvetkova K, Marvaud JC, Lambert T (2010) Analysis of the mobilization functions of the vancomycin resistance transposon Tn1549, a member of a new family of conjugative elements*.* Journal of bacteriology 192: 702-713.

78. Lyras D, Adams V, Lucet I, Rood JI (2004) The large resolvase TnpX is the only transposon-encoded protein required for transposition of the Tn4451/3 family of integrative mobilizable elements*.* Molecular microbiology 51: 1787-1800.

79. Merlin C, Springael D, Toussaint A (1999) Tn4371: A modular structure encoding a phage-like integrase, a Pseudomonas-like catabolic pathway, and RP4/Ti-like transfer functions*.* Plasmid 41: 40-54.

80. Springael D, Kreps S, Mergeay M (1993) Identification of a catabolic transposon, Tn4371, carrying biphenyl and 4-chlorobiphenyl degradation genes in Alcaligenes eutrophus A5*.* Journal of bacteriology 175: 1674-1681.

81. Carias LL, Rudin SD, Donskey CJ, Rice LB (1998) Genetic linkage and cotransfer of a novel, vanB-containing transposon (Tn5382) and a low-affinity penicillin-binding protein 5 gene in a clinical vancomycin-resistant Enterococcus faecium isolate*.* Journal of bacteriology 180: 4426-4434.

82. Dahl KH, Sundsfjord A (2003) Transferable vanB2 Tn5382-containing elements in fecal streptococcal strains from veal calves*.* Antimicrobial agents and chemotherapy 47: 2579-2583.

83. Shoemaker NB, Wang GR, Stevens AM, Salyers AA (1993) Excision, transfer, and integration of NBU1, a mobilizable site-selective insertion element*.* Journal of bacteriology 175: 6578-6587.

84. Shoemaker NB, Salyers AA (1988) Tetracycline-dependent appearance of plasmidlike forms in Bacteroides uniformis 0061 mediated by conjugal Bacteroides tetracycline resistance elements*.* Journal of bacteriology 170: 1651-1657.

85. Schmidt JW, Rajeev L, Salyers AA, Gardner JF (2006) NBU1 integrase: evidence for an altered recombination mechanism*.* Molecular microbiology 60: 152-164.

86. Shoemaker NB, Wang GR, Salyers AA (1996) The Bacteroides mobilizable insertion element, NBU1, integrates into the 3' end of a Leu-tRNA gene and has an integrase that is a member of the lambda integrase family*.* Journal of bacteriology 178: 3594-3600.

87. Shoemaker NB, Wang GR, Salyers AA (1996) NBU1, a mobilizable site-specific integrated element from Bacteroides spp., can integrate nonspecifically in Escherichia coli*.* Journal of bacteriology 178: 3601-3607.

88. Smyth DS, Robinson DA (2009) Integrative and sequence characteristics of a novel genetic element, ICE6013, in Staphylococcus aureus*.* Journal of bacteriology 191: 5964-5975.

89. Davies MR, Shera J, Van Domselaar GH, Sriprakash KS, McMillan DJ (2009) A novel integrative conjugative element mediates genetic transfer from group G Streptococcus to other {beta}-hemolytic Streptococci*.* Journal of bacteriology 191: 2257-2265.
